# Cash plus: exploring the mechanisms through which a cash transfer plus financial education programme in Tanzania reduced HIV risk for adolescent girls and young women

**DOI:** 10.1002/jia2.25316

**Published:** 2019-07-22

**Authors:** Audrey Pettifor, Joyce Wamoyi, Peter Balvanz, Margaret W Gichane, Suzanne Maman

**Affiliations:** ^1^ Department of Epidemiology Gillings School of Global Public Health University of North Carolina at Chapel Hill Chapel Hill NC USA; ^2^ Carolina Population Center University of North Carolina at Chapel Hill Chapel Hill NC USA; ^3^ National Institute of Medical Research Mwanza Tanzania; ^4^ Department of Health Behavior Gillings School of Global Public Health University of North Carolina at Chapel Hill Chapel Hill NC USA

**Keywords:** adolescents, HIV prevention, structural drivers, cash transfers, Africa, transactional sex

## Abstract

**Introduction:**

Cash transfers have been promoted as a means to reduce HIV risk for adolescent girls and young women (AGYW) in sub‐Saharan Africa. One of the main mechanisms whereby they are hypothesized to reduce risk is by deterring transactional sex. In this paper, we use qualitative methods to explore participant experiences, perspectives and reported behaviours of a cash transfer plus financial education programme among out of school, 15‐ to 23‐year‐old AGYWs in rural Tanzania with a focus on partner choice and transactional sex.

**Methods:**

We conducted 60 in‐depth interviews (IDIs) and 20 narrative timeline interviews with participants of the PEPFAR DREAMS Sauti/WORTH+ cash transfer programme between June 2017 and July 2018. Interviews were taped, transcribed and translated from Kiswahili to English. Transcripts were coded and analysed for key themes.

**Results:**

We found that participants in a cash transfer plus programme discussed behaviours that could reduce HIV risk through decreasing their dependence on male sex partners. There appeared to be two main mechanisms for this. One, young women discussed the cash transfer providing for basic needs (e.g. food, toiletries) which appeared to reduce their dependence on male sex partners who previously provided these goods (e.g. transactional sex). This experience was more pronounced among the poorest participants. Two, young women discussed how the financial education/business development aspect of the programme empowered them to refuse some sex partners; unmarried women discussed these experiences more than married women. Social support from family and programme mentors appeared to strengthen young women's ability to successful start businesses, produce income and thus be less dependent on partners.

**Conclusions:**

The cash transfer programme may have reduced AGYW engagement in transactional sex that occurred to meet basic needs (one form of transactional sex). The financial education/business development and mentorship elements of the programme appeared important in building AGYW agency, self‐esteem and future orientation which may support AGYWs in refusing unwanted sex partners. Future cash plus programmes should consider adding or strengthening financial education and job skills training, mentorship and future orientation to see stronger and perhaps sustainable outcomes for HIV prevention.

## 1 Introduction

Cash transfers are increasingly popular in Africa as part of many government social protection programmes with the aim of providing a social safety net for the most poor and vulnerable, allowing them to afford basic necessities such as food, shelter, education and medical care. In recent years, cash transfers have been identified as key strategy that could be leveraged to reduce HIV risk for adolescent girls and young women (AGYW) 1, 2.

There are a number of mechanisms through which cash transfers have been hypothesized to reduce HIV risk for AGYW including increasing school attendance, reducing stress and depression, and increasing hope for the future 3, 4. One of the main mechanisms is by reducing their economic dependency on men, in particular, reducing their need to engage in transactional sex (defined as non‐marital, non‐commercial sexual relationships motivated by an implicit assumption that sex will be exchanged for material support or other benefits) 5, 6, 7.

Data on the efficacy of cash transfers in reducing HIV risk for AGYW are mixed. Of five studies that have evaluated the impact of cash transfers on HIV risk in AGYW, the two that measured HIV incidence found no impact on incidence, one study found an impact on HIV prevalence, one study found a reduction in transactional sex and two found AGYW were less likely to report older partners 6, 7, 8, 9, 10. Some factors which may explain differences in results include the background level of poverty in the study area and among the target recipients, the amount of the transfer and frequency, gender and power norms for women, cultural constructions of materials goods, success and relationships, and the background level of social protection in the study area. These studies have highlighted the complexity of structural interventions and the importance that contextual factors play in impacting the effect of cash transfer programmes on HIV risk. In addition, they have raised questions as to whether cash transfers alone are sufficient for HIV prevention 11. In the prevention landscape, UNAIDS considers cash transfers as part of a combination package, addressing upstream/structural drivers of HIV risk 12.

Despite the mixed evidence, there are a number of cash transfer programmes in the field targeted to AGYW with the aim of reducing HIV risk. The President’s Emergency Plan For AIDS Relief (PEPFAR) DREAMS partnership (Determined, Resilient, Empowered, AIDS free, Mentored and Safe women), is a large HIV prevention initiative designed to reduce HIV risk among AGYW in 10 countries in Africa and includes the provision of cash transfers in some countries 13. In this paper, we use qualitative methods to explore participant experiences, perspectives and reported behaviours in a cash transfer plus financial education programme (Sauti/WORTH+) on out of school, 15‐ to 23‐year‐old AGYWs in rural Tanzania on HIV risk, in particular partner choice and transactional sex.

## 2 Methods

### 2.1 Parent study – Sauti project

We conducted our assessment with participants who participated in a DREAMS cash transfer plus programme which was implemented by the Sauti Project. Sauti Project is USAID‐funded programme that targets key and vulnerable populations with community‐based HIV prevention and reproductive health services. Cash transfers of TZS 70,000 (approximately USD 31) were provided to AGYW every three months for 18 months. AGYW who attended at least 10 hours of a behaviour change and communication (BCC) curriculum were eligible to participate in the cash transfer. Girls who completed the 10‐hour BCC curriculum, and were getting the cash, were offered the opportunity to participate in a small group financial literacy and individual savings and loan programme called WORTH+. The programme's theory of change was that AGYW would have more access to money thus being able to make healthier sexual decisions including reduced transactional sex, fewer older partners, fewer partners and more condom use. The cash transfer programme targeted AGYW aged 15 to 23 who were out of school.

### 2.2 Recruitment and study sites

We recruited female participants (n = 80) through community‐based social service organizations implementing the DREAMS project in northwest Tanzania. Participants were purposefully sampled from within selected study villages based on a vulnerability criteria (risky sexual behaviours) used by Sauti to identify adolescents for the DREAMS programme. We aimed to get an equal number of participants in the 15 to 19 and 20 to 23 age ranges and to sample more unmarried than married young women. Specifically, we recruited from Bulungwa village, Shinyanga municipal and Kahama. Locations are within three hours of each other and agriculture is the primary commerce. Bulungwa is more rural than the other two, with Shinyanga municipal being the district headquarters and Kahama having experienced rapid population boom with the existence of the Buzwagi gold mine at the edge of the town.

### 2.3 Data collection

#### 2.3.1 In‐depth interviews

We conducted 20 baseline interviews in June 2017 after the first cash transfer payments, and then again in June 2018. The 60 interviews included 20 follow‐up interviews with baseline participants and 40 new interviews. Interviews broadly assessed the impact of the cash transfer on AGYWs lives and how the cash may have influenced sexual decision‐making and partner choice.

#### 2.3.2 Narrative timeline interviews

We conducted narrative timeline interviews with 20 women 14, 15. These interviews asked AGYW to draw a timeline of sexual partnerships they had in the previous two years and to indicate the time when they received each cash transfer. Participants were then asked to discuss characteristics of each partner, including financial support, condom use, HIV testing and overall quality of the relationship. Interviewers probed about different qualities of each partner and how the cash may have impacted relationships.

All interviews were audio recorded and were conducted in Kiswahili by four trained female research assistants who were Swahili native speakers and experienced interviewers. Prior to the interviews, research assistants received refresher trainings on research ethics, the informed consent process, interview probing techniques and conducted pilot interviews with guides.

### 2.4 Analyses

Interviews were transcribed verbatim and translated from Kiswahili to English. Transcriptions were checked for quality by two of the study team members. We used applied thematic analysis to analyse data from all methods. Applied thematic analysis is a systematic, inductive process of sorting content by codes and conveying the meaning through themes. All transcripts were coded using Dedoose, a qualitative data analysis software program. For each method, an initial transcript was coded by four researchers, and then compared code by code across researchers. Following this global comparison, approximately 20% of the rest of the transcripts were quality checked by another researcher to ensure consistency in coding. We used coded transcripts to develop matrices 16 which included a summary of participant responses for each thematic category allowing for comparisons across interviews. The four researchers convened to discuss emergent themes focusing on the relationship between cash transfer receipt and HIV risk. In our discussion, we use the following terms to describe how common each theme was: a few means not that many people discussed the issue, some means that more than a few but less than half discussed the issue and many/most means more than half discussed the issue.

### 2.5 Ethical review

We obtained Institutional Review Board (IRB) approval from The University of North Carolina and the National Institute for Medical Research in Tanzania. Informed consent was obtained prior to any interview or focus group session.

## 3 Results

**Figure 1:**
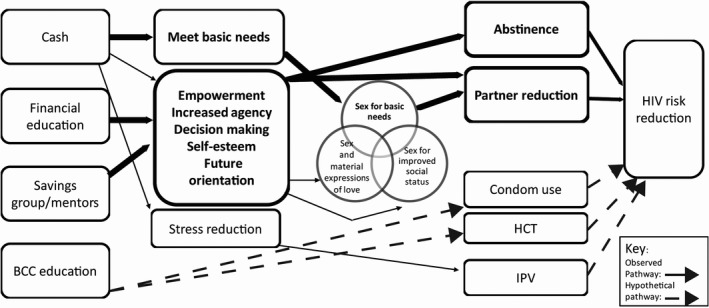
**Conceptual framework on pathways through with the Sauti/WORTH+ cash transfer programme reduced HIV risk for AGYW.** Solid lines, observed pathways, display pathways discussed by participants in the interviews. Dotted lines, hypothetical pathways, are those that the literature or other conceptual frameworks have endorsed. The transactional sex concepts are drawn from the conceptual framework by Stoebenau *et al*. Words and lines in bolder text are related to items that were more frequently discussed by participants in the interviews presented here. HCT, HIV counselling and testing; IPV, intimate partner violence.

Table 1 presents the demographic profile of the young women who participated in these interviews. Here, we present key themes on possible mechanisms through which cash transfer programmes may operate to reduce HIV risk for young women. The conceptual framework was developed after analysis of the data and is informed by our interpretation of the data and prior literature on how cash transfers reduce HIV risk (Figure 1). Solid lines are pathways discussed by young women in the interviews, dotted lines are theorized pathways that other literature and cash transfer studies have observed but we did not observe in this study. Below, we explore components of the conceptual framework as they relate to partner selection and provide information generated from the interviews to expand on the themes identified.

**Table 1:** Demographic characteristics of AGYW participants taking part in interviews

Variables	Total population
	% (n = 80)
Age in years	
15 to 19	47.5 (38)
20 to 24	52.5 (42)
Education	
Primary school or less	53.8 (43)
Some secondary school	12.5 (10)
Secondary school completed or more	33.8 (27)
Marital status	
Not married	65 (52)
Married	35 (28)
Children	
No	50 (40)
Yes	50 (40)
Residence	
Bulungwa	28.8 (23)
Kahama	28.8 (23)
Shinyanga	42.5 (34)

### 3.1 Programme design played a key role in programme effects

The design of the cash transfer programme had an important influence on how cash was used and thus the potential of the programme to influence partner selection and thus HIV risk. The aim of the programme was to provide young women with money so that they could develop businesses, earn their own income and thus be less dependent on men. The vast majority of AGYW internalized this messaging as illustrated in the quote below:

“They advised us to open our business/plans, not to be influenced by men by whom we (Inhalation), for example, having needs for money, do not go to request a man but you satisfy your own needs” (17 years old, not married, no child, ID21N)

I: Mhh did they tell you why you were given the money for?

R: For the purpose of uplifting us so that we don't depend on men (21 years old, married, 1 child, ID16L)

### 3.2 Cash helped meet basic needs for the most vulnerable AGYW

AGYW talked about the cash addressing some of the immediate consequences of poverty such as food insecurity and access to basic needs (e.g. sanitary pads, soap). Some AGYW talked about how there was no money in their home for food at times and how the cash transfer allowed them to buy food. Thus, the cash appears to have helped provide for basic needs for some young women, especially the poorest.

“Eee sometimes when they [family] don't have money at home and there's no food, if I have money [the cash transfer] then I take care of them at home, I can buy flour, rice” (19 years old, not married, one child, ID18 L)

AGYW who came from homes that were more financially stable discussed being able to use the money to develop or grow a business or spend on their own needs than young women who were more poor. In fact, for young women who were financially better off, they talked about family providing extra money to develop a business or grow an existing business or attend training courses which helped AGYW succeed in earning income.

“As for me when I requested her (mother) to help me buy a sewing machine, she accepted and told me she is in the process of inquiring so that we can buy.” (18 years old, not married, no child, ID1N)

For those who were very poor, some AGYW talked about having to spend the money for basic needs or emergencies instead of investing in starting a business.

“For me honestly when I receive the first installment I did not invest it into business. I only bought clothes and food” (16, unmarried, 1 child, ID101N)

### 3.3 Cash covered AGYW basic needs which reduced reliance on male partners

Girls discussed that the cash allowed them to buy small things that they could not previously afford prior to the cash transfer. Many unmarried young women talked about how the cash reduced their need to seek out male partners to cover their basic needs. The poorer young women talked about this more than young women who appeared better off financially.

“It [the cash transfer] helped us a lot to overcome the temptations [seeking out men] and to be able to afford our small needs … Because at first I was thinking on how to get my own money, but after getting this cash transfer it has reduced my stress on where to get the money… what will I do… but after receiving it has reduced it in one way or another” (18 years old, unmarried, no children, ID103N)

“Before I used to think of men…You find that I don't have any money…You find that I think about men a lot. But for now with the money being sent… I have opened a business. So now those thoughts are no longer in my mind.” (21 years old, married, 1 child, ID8L)

Some young women when asked directly about how the cash influenced partner selection reported that it did not. This feeling was predominantly expressed by women who were already in committed relationships and younger women who had never been in a relationship.

“But like me it has changes my behavior to the extent that it has changed in terms of life and business wise..But not in partners” (24 years old, married, 1 child, ID16N).

A few married women did talk about engaging in extramarital relationships to get additional money to support their families and that the cash meant they did not have to seek out other partners to provide financial support; however, most transactional sex appeared to take place among unmarried women.

### 3.4 Entrepreneurial skills enhanced future aspirations

Some young women talked about how the cash transfer coupled with business development skills supported stronger future aspirations. A few AGYW talked about going for job training or buying assets such as livestock or land that would provide income or financial security beyond the limited scope of the cash payments, showing a focus on the future.

“When I received a cash transfer for the first time I went to a certain woman with a sewing machine and begun to learn how to sew and the second time [second payment] I also used it there… The third time when I received I was given some money at home so I added to it and bought my own sewing machine” (18 years old, not married, no children, ID7N)

### 3.5 Social support enhanced young women's entrepreneurial success

Young women talked about how social support from family and programme mentors helped them succeed in meeting their business goals. Many AGYW talked about the importance of family supporting them to meet their financial goals, and providing advice related to the cash and staying on track with meeting business and financial goals.

“I felt happy because after telling her [sister] that am doing a certain business then she advised me on how to use that money so I just knew she's still with me … until now she still advises me”(24 years old, not married, 1 child, ID9N)

Male partner support appeared common for married but not unmarried women. This seemed to be because married women lived with their husbands and made financial decisions with them. In most cases, married women talked about men being supportive of the cash as it was used for the household. Most unmarried young women reported not telling male partners about the cash because they felt the relationship was not serious enough to warrant discussing personal finances, they were worried they would lose financial support from partners or due to lack of trust of partners.

Many unmarried AGYW talked about stress reduction in their families from not having to ask mothers or other family members for money to pay for small, personal items (e.g. sanitary pads, lotion, oil). A few married women talked about how the cash reduced financial stress in the home which reduced tension and potentially violence from partners which ultimately may have improved relationship quality.

As part of the cash transfer programme, AGYW were encouraged to participate in savings groups and had mentors who advised them on how to develop a business and save. AGYW talked about the savings clubs providing financial support when they needed it (they could ask the group for money instead of men) and that it provided support in growing their business.

“My views about the group since I have seen its benefits. First, I can say that it helps when you're keeping your savings there because you can take a loan and do business on something that you know you'll make a profit and return the loan you took from the group. By doing so it eliminates [sexual] temptations because you may say that you'd want to do a certain business and maybe the starting capital is 100,000, and you do not have that money and maybe your parents also cannot help and you meet someone on the street and someone [sex partner] offers to give you 150,000. It helps to avoid such temptations.” (19 years old, not married, no children, ID14NT)

Young women talked about how programme mentors helped keep AGYW on task with the development of a business and savings. Some AGYW talked about how they spent the first payment on things not related to building a business but through guidance from programme mentors they were able to spend future payments on business development.

### 3.6 Cash and business experience empowered AGYW which enabled them to refuse unwanted sex partners and abstain from sex

An important outcome of the cash and entrepreneurial training that young women talked about was that it allowed them to take care of themselves and meet their own small needs without having to depend on anyone else, be it family members or partners, which created a sense of empowerment, agency, self‐reliance and pride.

“Because I wanted to reach my goals as a young person so I did not need to have a partner. After the organisation started sending me money I had faith in myself and believed I did not need anyone.” (19 years old, married, no children, ID29N)

Some young women discussed how this empowerment and self‐esteem/pride that the programme fostered gave them the agency to say no to men who might offer them money. Financial independence empowered some young women to say no to partners they were not interested in.

“As in pride as in you have your own money as in even if someone talks to you, you really don't have time for them as in you just don't care…If you meet up with him and he tells you something then you can tell him to leave you alone but it's hard if you don't have money” (24 years old, married, children, ID16N).

“We first get the money project and engage ourselves so a man just can't come from nowhere and I just love him and right now we are proud of something we also have money so a man can't stress me and I also I can look for that man who has a higher earning than me.” (24 years old, not married, children, ID9N)

For the few younger women who had not ever had sex, they talked more about the programme helping them to abstain from sex while for AGYW who had already had sex they talked more about refusing unwanted partners, thus reducing partner number. A few young women also talked about how they were too busy running a business and thinking about making money to have time for men. Overall, there was minimal discussion of condom use or other HIV prevention behaviours such as testing as a direct result of the cash transfer.

## 4 Discussion

In this qualitative study of a cash plus financial education programme among out of school AGYW in rural Tanzania, we found that provision of the cash transfer helped some young women meet basic needs which appeared to reduce transactional sex motivated by the need for food and basic goods. Importantly, the cash transfer plus financial education and savings groups appeared to increase AGYWs agency and self‐esteem, empowering them to refuse some male sex partners, possibly reducing HIV risk.

One of the main hypotheses of how cash transfers may reduce HIV risk for AGYW is by reducing their financial dependence on men thereby reducing engagement in transactional sex 6, 7. We found that the Sauti/WORTH+ programme may have helped young women refuse sex with men for basic needs, likely the most poor. The assumption that small cash transfers will reduce transactional sex among AGYW relies on the assumption that transactional sex is motivated by poverty. However, using the conceptual framework by Stoebenau *et al*. describing motivators for transactional sex, sex for basic needs is only one reason why young women engage in transactional sex 5. Depending on the context and background poverty level of an area, the ability of a cash transfer to reduce transactional sex may depend on the proportion of young women engaging in transactional sex to obtain basic needs. It seems less likely that a small cash transfer will have a strong impact on other motivations for engaging in transactional sex including desire for social status and sex and material expressions of love. Thus, we posit that cash transfer programmes will reduce HIV risk by helping provide for basic needs and this may have had a modest impact on one element of risky transactional sex, sex for basic needs thus reducing partner number (Figure 1).

A second and perhaps more important pathway through which a cash plus financial education/business development programme could reduce AGYW's HIV risk is by increasing their agency and self‐esteem (Figure 1). For many young women in this programme, this was the first time they had access to their own money to spend how they pleased. Elements of the programme that focused on building and running a business, making money and saving resulted in AGYW gaining courage to do new things, greater decision‐making abilities and self‐confidence they could provide for themselves and meet their future goals independent of a male partner. The business development and savings component appeared to help some young women think more about their future resulting in them saving to buy land, livestock or assets for their business. This benefit is something that cash alone programmes likely would not provide.

The confidence and agency gained from the programme by AGYW appeared to translate to some AGYW being able to have greater choice over sex partners. The empowerment agency and self‐esteem gained appeared to have a direct impact on partner reduction or delayed coital debut/abstinence (Figure 1). It also may have had an indirect and small impact on transactional sex that is motivated by social status, where girls may have sex to obtain “commodities of modernity” which allow them to gain social status with peers 5. Through improved self‐esteem and future aspirations, it is possible AGYW may find confidence from within themselves and not from material goods. The other element of transactional sex addresses the interconnectedness of love and money in romantic relationships 17. Traditional gender norms in many settings expect men to provide for women/family and women in turn reciprocate by providing sex and domestic services. We argue that women's increasing participation in provision of household needs may start to challenge traditional gender norms of male provision. The implication of these gradual shifts in gender roles is unclear and further research would be necessary to understand the effect on women's economic and sexual empowerment.

While the Sauti/Worth+ programme focused on out of school AGYW, it did not exclude married young women. However, the impact of the cash plus programme in reducing HIV risk for married women in this programme seemed minimal given most risk likely comes from male partners/husbands. It is unclear that including married women in cash transfer programmes to reduce HIV risk will have a major impact.

Social support from family and the programme mentors and participants appeared important in helping AGYW be more successful in achieving financial goals. The programme design of WORTH+ hypothesized that savings groups would provide social support from fellow AGYW. Importantly, programme mentors who lead the savings groups appeared important in helping young women set up businesses and stay on track with business development when they were struggling. It is possible that future programmes would benefit from further strengthening the mentorship piece. Finally, family and parents, appeared to play an important role in the financial and business decisions AGYWs contributing to their success and their role should be considered in future programming.

Sustainability of programme effects once cash transfer programmes end is an important consideration. The Sauti/WORTH+ project provided six payments to AGYW over 18 months. This is a relatively short period of time to build benefits that can extend beyond the programme end. For young women who were able to build a sustainable business, acquire job skill training or purchase assets, it is possible that programme effects may extend beyond the end of the programme while for those who did not and were reliant on male partners, it seems unlikely that any programme effects on partner reduction and transactional sex will be maintained.

The current thinking in the cash transfers for HIV prevention field is that cash alone will likely not reduce HIV risk for AGYW significantly 11, 18. Our findings support this view. This has led to the advocacy for “cash plus” programmes meaning that cash transfers should be packaged with other services, including mentorship, financial education, and health services. While the impacts of these programmes are still to be seen, from this qualitative exploration, it appears that future cash plus programmes should consider including financial education and savings groups and focus on unmarried AGYW. A key message in the Sauti/WORTH+ programme was that it was to make AGYW less financially dependent on men; AGYW internalized this message. Future programmes may want to consider similar clear, strong programme messaging. Adding programming around future orientation may also be important so AGYW can use the cash to advance long‐term savings and business goals. In addition, strengthening AGYW mentorship could play a critical role in helping AGYW achieve financial and future life goals. Programme mentors who can provide training in key job and life skills could help AGYW transition from adolescence into adulthood more successfully.

### 4.1 Limitations

There are a number of limitations of this study. As this is qualitative study, the purpose was to explore experiences with a cash transfer programme and possible influences on partner selection and thus we cannot draw any conclusions about the programme impact on HIV. Second, the Sauti/WORTH+ programme had strong messaging around reducing young women's reliance on male partners; thus, it is possible that social desirability bias influenced AGYW responses even though our research team were independent of the Sauti team. Along these lines, girls selected to participate in the interviews may not be representative of all girls in the programme. Third, few young women discussed how the programme influenced HIV prevention modalities such as condom use or HIV testing; however, we did not ask direct questions about this.

## 5 Conclusions

Overall, we found that out of school AGYW in rural Tanzania who participated in a cash plus financial education programme discussed some possible pathways through which the programme may have reduced dependence on male partners. The pathways discussed included the cash providing for AGYWs basic needs that they previously acquired from male partners and also through increased empowerment, agency and self‐esteem acquired from the financial education, savings groups and business development aspects of the programme. Future cash plus programme should consider including financial education/savings groups, mentorship and social support for AGYW, and more training on future goals and aspirations.

## Competing interests

The authors have no competing interests to declare.

## Authors’ contributions

Audrey Pettifor conceptualized the study, developed the protocol and study guide, reviewed the transcripts, analysed the data, identified the themes and wrote the paper. Joyce Wamoyi conceptualized the study, developed the protocol and study guide, oversaw the field work, reviewed the transcripts, analysed the data, identified the themes and edited the paper. Peter Balvanz provided the study oversight and implementation, coded the transcripts, analysed the data, identified the themes and edited the paper. Margaret Gichane coded the transcripts, analysed the data, identified the themes and edited the paper. Suzanne Maman conceptualized the study, developed the protocol and study guide, reviewed the transcripts, analysed the data, identified themes and edited the paper.

## References

[1] Pettifor A , MacPhail C , Nguyen N , Rosenberg M . Can money prevent the spread of HIV? A review of cash payments for HIV prevention. AIDS Behav. 2012;16(7):1729–38. 22760738 10.1007/s10461-012-0240-z PMC3608680

[2] Heise L , Lutz B , Ranganathan M , Watts C . Cash transfers for HIV prevention: considering their potential. J Int AIDS Soc. 2013;16:18615. 23972159 10.7448/IAS.16.1.18615 PMC3752431

[3] Handa S , Palermo T , Rosenberg M , Pettifor A , Halpern CT , Thirumurthy H . How does a national poverty programme influence sexual debut among Kenyan adolescents? Glob Public Health. 2017;12(5):617–38. 26853950 10.1080/17441692.2015.1134617 PMC4976080

[4] The Transfer Project . Ujana Salama: cash plus model on youth well‐being and safe, healthy transitions. Chapel Hill, NC: Carolina Population Center; 2018.

[5] Stoebenau K , Heise L , Wamoyi J , Bobrova N . Revisiting the understanding of “transactional sex” in sub‐Saharan Africa: a review and synthesis of the literature. Soc Sci Med. 2016;168:186–97. 27665064 10.1016/j.socscimed.2016.09.023

[6] Cluver L , Boyes M , Orkin M , Pantelic M , Molwena T , Sherr L . Child‐focused state cash transfers and adolescent risk of HIV infection in South Africa: a propensity‐score‐matched case‐control study. Lancet Glob Health. 2013;1(6):e362–70. 25104601 10.1016/S2214-109X(13)70115-3

[7] Pettifor A , MacPhail C , Hughes JP , Selin A , Wang J , Gómez‐Olivé FX , et al. The effect of a conditional cash transfer on HIV incidence in young women in rural South Africa (HPTN 068): a phase 3, randomised controlled trial. Lancet Glob Health. 2016;4(12):e978–88. 27815148 10.1016/S2214-109X(16)30253-4 PMC5626439

[8] Baird SJ , Garfein RS , McIntosh CT , Özler B . Effect of a cash transfer programme for schooling on prevalence of HIV and herpes simplex type 2 in Malawi: a cluster randomised trial. Lancet. 2012;379(9823):1320–9. 22341825 10.1016/S0140-6736(11)61709-1

[9] Handa S , Peterman A , Huang C , Halpern C , Pettifor A , Thirumurthy H . Impact of the Kenya cash transfer for orphans and vulnerable children on early pregnancy and marriage of adolescent girls. Soc Sci Med. 2015;141:36–45. 26246032 10.1016/j.socscimed.2015.07.024 PMC4659857

[10] Abdool Karim Q , Leask K , Kharsany A , Humphries H . Impact of conditional cash incentives on HSV‐2 and HIV prevention in rural South African high school students: results of CAPRISA 007 cluster randomized trial. International AIDS Conference. Vancouver, Canada; 2015.

[11] Cluver LD , Orkin FM , Boyes ME , Sherr L . Cash plus care: social protection cumulatively mitigates HIV‐risk behaviour among adolescents in South Africa. AIDS. 2014;28 Suppl 3:S389–97. 24991912 10.1097/QAD.0000000000000340

[12] UNAIDS . Fast‐tracking combination prevention. Geneva; 2015.

[13] Presidents Emergency Fund for AIDS Relief (PEPFAR) . DREAMS Core Package of Interventions Summary https://www.pepfar.gov/documents/organization/269309.pdf

[14] Goldenberg T , Finneran C , Andes KL , Stephenson R . ‘Sometimes people let love conquer them’: how love, intimacy, and trust in relationships between men who have sex with men influence perceptions of sexual risk and sexual decision‐making. Cult Health Sex. 2015;17(5):607–22. 25465292 10.1080/13691058.2014.979884 PMC4492250

[15] Goldenberg T , Finneran C , Andes KL , Stephenson R . Using participant‐empowered visual relationship timelines in a qualitative study of sexual behaviour. Glob Public Health. 2016;11(5–6):699–718. 27092985 10.1080/17441692.2016.1170869

[16] Miles MB , Huberman A . Matrix displays: some rules of thumb. Qualitative data analysis: an expanded sourcebook. Thousand Oaks: Sage Publications; 1994.

[17] Majola S . Love, Money, and HIV becoming a modern African woman in the age of AIDS. Oaklandm, CA: University of California Press; 2014.

[18] Cluver LD , Orkin FM , Yakubovich AR , Sherr L . Combination social protection for reducing HIV‐risk behavior among adolescents in South Africa. J Acquir Immune Defic Syndr. 2016;72(1):96–104. 26825176 10.1097/QAI.0000000000000938 PMC4839503

